# CD6, a Rheostat-Type Signalosome That Tunes T Cell Activation

**DOI:** 10.3389/fimmu.2018.02994

**Published:** 2018-12-18

**Authors:** Carine M. Gonçalves, Sónia N. Henriques, Rita F. Santos, Alexandre M. Carmo

**Affiliations:** ^1^Instituto de Investigação e Inovação em Saúde, Universidade do Porto, Porto, Portugal; ^2^Instituto de Biologia Molecular e Celular, Porto, Portugal; ^3^Instituto de Ciências Biomédicas Abel Salazar and Faculdade de Ciências, Universidade do Porto, Porto, Portugal

**Keywords:** inhibitory receptors, CD5, CD6, signalosome, T lymphocytes

## Abstract

Following T cell receptor triggering, T cell activation is initiated and amplified by the assembly at the TCR/CD3 macrocomplex of a multitude of stimulatory enzymes that activate several signaling cascades. The potency of signaling is, however, modulated by various inhibitory components already at the onset of activation, long before co-inhibitory immune checkpoints are expressed to help terminating the response. CD5 and CD6 are surface glycoproteins of T cells that have determinant roles in thymocyte development, T cell activation and immune responses. They belong to the superfamily of scavenger receptor cysteine-rich (SRCR) glycoproteins but whereas the inhibitory role of CD5 has been established for long, there is still controversy on whether CD6 may have similar or antagonistic functions on T cell signaling. Analysis of the structure and molecular associations of CD5 and CD6 indicates that these molecules assemble at the cytoplasmic tail a considerable number of signaling effectors that can putatively transduce diverse types of intracellular signals. Biochemical studies have concluded that both receptors can antagonize the flow of TCR-mediated signaling; however, the impact that CD5 and CD6 have on T cell development and T cell-mediated immune responses may be different. Here we analyze the signaling function of CD6, the common and also the different properties it exhibits comparing with CD5, and interpret the functional effects displayed by CD6 in recent animal models.

## Introduction

Antigen-specific T cell activation is triggered by the T cell receptor (TCR) recognition of a cognate peptide presented by antigen presenting cells (APC), but it is overall controlled by a plethora of other cell surface receptors that either increase or repress the strength of the signals, the combination of which determines the outcome of T cell-mediated responses. Most of the receptors do not contain intrinsic enzymatic activities so their function relies on the establishment of interactions with signaling effectors, and also on an appropriate localization where they can exert their role, determined extracellularly by the binding to specific ligands expressed on the APC and intracellularly through connecting with the cytoskeleton.

Inhibitory co-receptors, such as the immune checkpoints Programmed cell death protein 1 (PD-1), Cytotoxic T-lymphocyte-associated protein 4 (CTLA4), T cell immunoreceptor with Ig and ITIM domains (TIGIT), Lymphocyte-activation gene 3 (LAG-3), and B- and T-lymphocyte attenuator (BTLA) are crucial to halt the progression or to terminate cell activation once they become expressed, given that they are strongly induced upon activation (1–5). They exert their inhibitory effect intracellularly through very limited and defined interactions utilizing ITIM, ITSF, or other sequences of their cytoplasmic tails that are bound by inhibitory enzymes, typically serine/threonine or tyrosine phosphatases (6–10). Moreover, some of these inhibitory co-receptors also interfere with co-stimulatory receptors through extracellular competition for the same ligands (11–14).

A different class of inhibitory receptors that can be active immediately following the triggering of the TCR and thus modulate T cell signaling at the onset of activation is exemplified by CD5: constitutively expressed on nearly all T cell subsets, including naïve and non-activated cells (15, 16), and loosely interacting with the TCR/CD3 complex (17), CD5 is able to modulate responses concomitantly with TCR triggering (18, 19). The expression of CD5 can increase significantly upon thymocyte and mature T cell activation such that the potency of the inhibition is proportionally adjusted in accordance to the affinity of TCR recognition of peptide/MHC and TCR-dependent signaling intensity (20, 21). This indicates that the variable levels of CD5 expression are important to counteract the strength of TCR signaling (22).

CD6 shares with CD5 many genetic, structural, and functional characteristics, among them the capacity to interact with the TCR/CD3 complex, to be tyrosine-phosphorylated and activated upon TCR triggering, and slightly increases its surface expression upon T cell activation (23–26). However, some differences between the kinetics of expression of CD5 and CD6 during thymocyte ontogeny and selection and T cell subset polarization as well as apparent different requirements for ligand binding suggest that CD5 and CD6 may have non-coincident roles during thymocyte development, T cell activation and immune responses. Nevertheless, in cellular systems it was also shown that the strength of activation correlates inversely with the expression of CD6 (27). Therefore, similarly to CD5 and distinctive from the immune checkpoints that can shut down activation at later stages, CD6 may be a rheostat-type regulator of activation, fine-tuning the response depending on the strength of the antigenic challenge.

## CD6 Is a Hub for the Assembly of Stimulatory and Inhibitory Effectors

At the time of the cloning of their genes and initial functional characterization of the proteins, CD5 and CD6 were regarded as co-stimulatory receptors that amplified TCR-dependent activation (28–31). In the case of CD5, the function of the molecule was soon after revised following the development of mice with a disrupted *Cd5* gene (18). The lack of similar definitive models addressing the role of CD6 *in vivo* until very recently delayed significantly the progress on CD6 research, and caused that the knowledge on the function of CD6 is still lagging considerably behind.

There are many common aspects in the biochemical behavior of CD5 and CD6 and in fact they can interact with each other in non-activated T cells (32, 33). Upon antigen recognition and T cell-APC conjugation, both receptors localize at the center of the immunological synapse (33). In contact with the TCR/CD3 signaling machinery, CD5 and CD6 are very rapidly phosphorylated on tyrosine residues (19, 24), presumably by the SRC-family kinase LCK, with the concomitant docking of intracellular mediators that contain SH2 domains, semi-autonomous conserved structural domains that bind to phosphorylated tyrosine residues. The net contribution of either CD5 and CD6 appears to be inhibitory, given that cells that lack any of the receptors are significantly more responsive to antigenic or mitogenic stimulation (22, 34). However, the number and diversity of effectors that associate with CD5 and/or CD6, depending or not on tyrosine phosphorylation, would not give an obvious idea of the repressive potential of the receptors, given that many interacting partners are effectively protein tyrosine kinases that are normally associated with signaling progression. These include LCK, FYN, ZAP70, and additionally in the case of CD6, the TEC-family kinase ITK (32, 35–37).

Perhaps this aggregation of kinases at the cytoplasmic tail of CD5 and CD6 explains the behavior observed in their initial characterization when either receptor, when triggered together with the TCR/CD3 complex with monoclonal antibodies, amplified the activation signals originated at the TCR complex. Notwithstanding this possibly artifactual contribution to activation determined by the *in vitro* experimental design, it is also possible that the kinases may actually contribute to positive signaling via CD5 and CD6 in very defined contexts, thus explaining the dual function that has been many times attributed to CD6 and occasionally to CD5.

**Figure 1:**
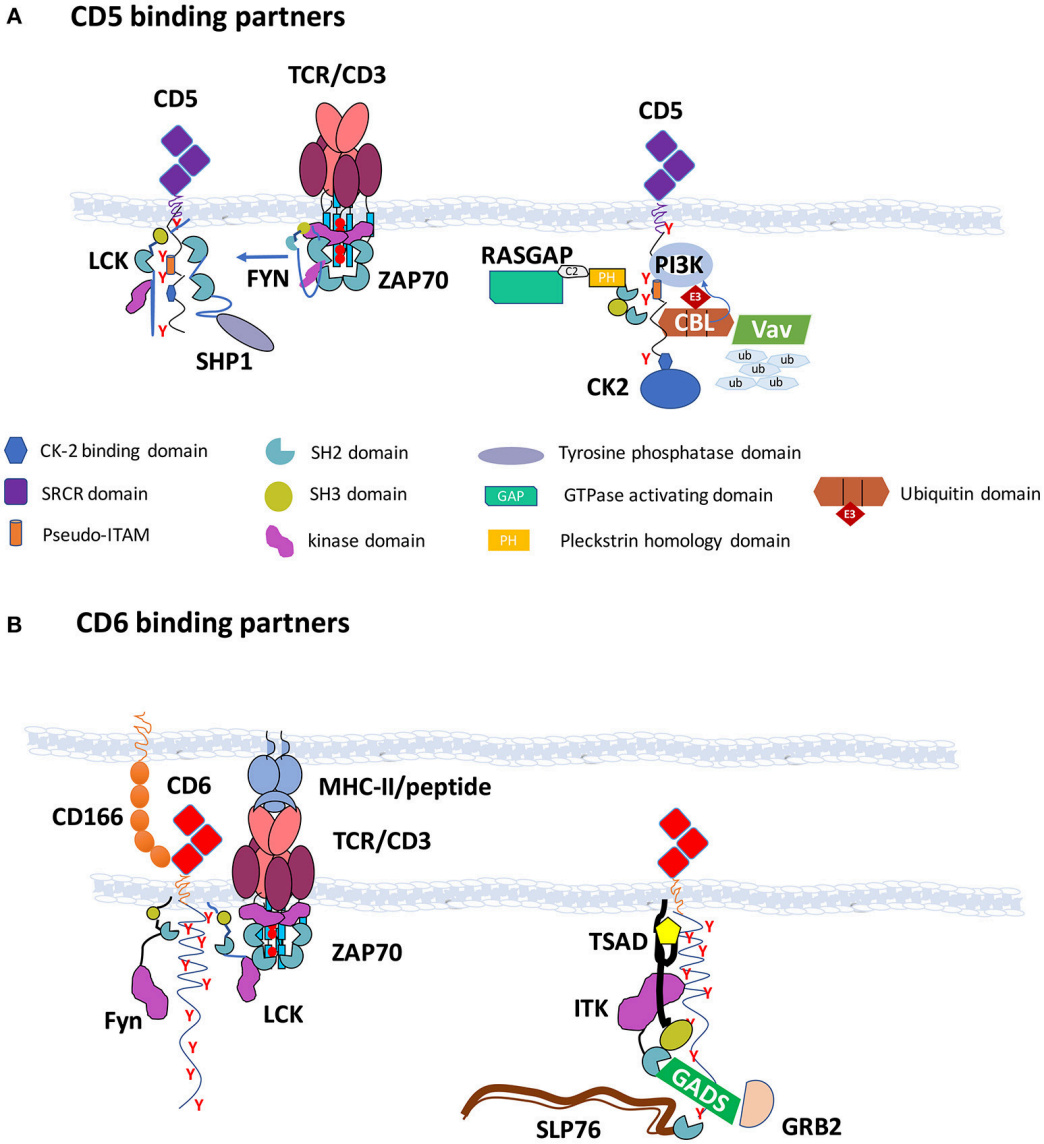
CD5 and CD6 are hubs for the assembly of effector enzymes and adaptors—**(A)** CD5 binding partners: CD5 contains in its cytoplasmic tail four tyrosine residues, of which three (Y453, Y465, and Y487) are believed to be phosphorylated upon TCR triggering and can bind the SH2 domains of LCK, RASGAP, CBL, CBLB, SHP1, and PI3K. Recruitment of CBL to the C-terminal region of CD5 is important for the ubiquitylation and degradation of several substrates following TCR engagement, including VAV. CK2 is also able to bind to the cytoplasmic tail of CD5 through other mechanisms. The interaction with FYN is also not dependent on tyrosine phosphorylation. CSK associates with the CD5 signalosome possibly through the cooperation with PAG, CBL, or CBLB. CD5 is represented in duplicate to accommodate all binding partners; **(B)** CD6 binding partners: CD6 contains in its cytoplasmic tail nine tyrosine residues that when phosphorylated can dock the SH2 domains of SLP76, TSAD, GADS, GRB2, and SHP1. The interactions with LCK, FYN, ZAP70, and ITK were not shown to be dependent on SH2 domain binding to phosphotyrosine residues, but ITK may be recruited through its association with TSAD. CD6 binds through the C-terminal sequence to the PDZ domains of syntenin. The CD6 signalosome is depicted in the right. Structures are not drawn to scale.

CD5 contains four tyrosine residues on its cytoplasmic domain, that when phosphorylated constitute putative sites for the docking of SH2 domain-containing cytoplasmic molecules. Tyrosine 402 is close or even buried within the plasma membrane and therefore it is disputable whether it can actually be phosphorylated. Nonetheless, the remaining tyrosine residues of CD5, when phosphorylated, have been for a long time shown to bind to the tyrosine kinase LCK (35), the tyrosine phosphatase SHP1 (38, 39), the ubiquitin ligases CBL and CBLB (40, 41), the GTPase activating protein for RAS (RASGAP) (40) and the lipid kinase PI3K (42), while the associations of CD5 with the protein kinases FYN and ZAP70 have not been shown to be direct (Figure 1A).

CD6 possesses possibly the longest cytoplasmic tail of the known receptors of leukocytes, containing amongst other signaling motifs nine tyrosine residues. However, fewer than expected interactions of CD6 with SH2 domain-containing effectors have been reported to date, possibly because research on CD6 function has been performed less systematically than that on CD5 (Figure 1B). Moreover, most of the disclosed interactors of CD6 seem to be more related to activation pathways rather than to repression mechanisms, including the tyrosine kinases LCK, FYN, ZAP70, and ITK that were shown to be associated with CD6 but not confirmed to be dependent on phosphotyrosine-SH2 domain binding. Additionally, the adaptors SLP76 (27), TSAD (43), GADS, and GRB2 (44), that have established roles in T cell activation have been shown to bind to the two most carboxyl-terminal phosphotyrosines of CD6. CD6 also binds to the scaffolding protein syntenin-1, but the interaction is likely mediated by the tandemly arranged PDZ domains of syntenin-1 (45).

Interestingly, some of the papers that reported the phosphoprotein-dependent molecular interactions of CD6 described the coincident finding that the same molecules could also dock onto the phosphotyrosine sites of Linker for activation of T cells (LAT), a membrane-bound adaptor of the main axis of the TCR-mediated pathway. In an unbiased *in vivo* proteomics screening, Roncagalli et al. described the LAT-independent association of SLP76 to CD6 (46), while Hem et al. showed that TSAD bound to both LAT and CD6 (43). Given that additionally GRB2 and GADS are well-established binders of LAT (47), it emerges that CD6 displays some characteristics of membrane-bound adaptors, such as LAT and Phosphoprotein associated with glycosphingolipid-enriched microdomains 1 (PAG) in that it contains multiple tyrosine residues that once phosphorylated can couple to a diverse set of signaling effectors, possibly feeding onto various, convergent or divergent, signaling pathways (48).

Therefore, and much like LAT and PAG, CD6 seems to constitute a signalosome that assembles many different enzymes and adaptors that can impact on signal propagation in different pathways and potentially with divergent outcomes. *Lat* knockout mice, however, show a very different behavior than those deficient of CD6 (described later in this paper), displaying a block in the development of thymocytes at the double negative stage and a complete absence of mature T cells (49). This established LAT as a crucial adaptor for T cell signaling leading to lymphocyte differentiation and also of T cell activation. On the other hand, mice with a disrupted *Pag* gene, much similarly to *Cd6*-deficient but also to *Cd5*-deficient animals, have no overall differences in total numbers of T cells than wild-type mice; and effector T cells, although not naïve, are more prone to activation upon TCR targeting (50). PAG is essential for the phosphotyrosine-dependent docking of the protein tyrosine kinase CSK, a major inhibitory enzyme of T cell activation that phosphorylates the inhibitory carboxyl-terminal tyrosine residues of LCK and FYN, inactivating these kinases (51, 52). Interestingly, CD5 seems to be a relevant alternative docking receptor for CSK, and in fact PAG and CD5 may cooperate in the inhibition of FYN (36, 41). Overall, it appears that while LAT is a hub for the assembling of positive effectors, CD5 as well as PAG can be viewed as inhibitory signalosomes. Whether CD6 is an activating, inhibitory, or multi-purpose scaffolding transmembrane receptor is still under debate.

Given that CD6, besides being structurally very similar to CD5, shares with it many features, such as profile of expression and functional roles in the biochemical repression of T cell activation, it would be expected or at least plausible that, similarly to CD5, it could couple to analogous signaling inhibitory partners. A recent report has in fact described the interaction of CD6 with the protein phosphatase SHP1 (26), constituting this the first solid biochemical evidence that CD6 can couple to inhibitory signaling.

## Can Ligand Binding and Signaling Inhibition be Uncoupled Events?

Unlike PAG or LAT, CD6 contains structured ectodomains that are suitable to establish interactions with extracellular ligands. CD6 binds to CD166, widely expressed in many cell types and tissues (53, 54). and recently CD318 was identified as an alternative ligand in cells derived from human thymus, skin, synovium, and cartilage (55, 56). However, there is no absolute requirement for CD6 to bind to ligands to be able to exert its inhibitory function (34). This is a characteristic common to CD5 (57). Therefore, these two receptors can be general attenuators of TCR-mediated signaling independent of any mechanical effects of ligand binding or of any particular membrane localization. So, if there can be a functional uncoupling between these two features of the molecules, what may be the role of the ectodomains and the consequences of binding to ligands?

During thymocyte development, the expression of CD6 increases steadily from double negative (DN) to double positive (DP) and to single CD4^+^ or CD8^+^ thymocytes, decreasing then slightly in the negatively selected single CD4^+^ or CD8^+^ thymocytes just before thymic emigration, and to mature T cells (58). The increasing expression of CD6 favors the interaction with CD166, highly expressed on thymic epithelium, possibly providing anti-apoptotic signals and also increasing the adhesion of thymocytes to thymic epithelial cells. However, this signal tuning-related variation of expression is a general characteristic and not a differentiation feature, i.e., thymocytes are exposed to APCs that all express the same putative CD6 ligands and thus there should be no distinctive outcome in selection or subset polarization between different thymocytes based solely on whether or not CD6 binds to its ligand.

The scenario can be remarkably different regarding the activation of mature T cells, though. The interaction between CD6 and CD166 is one of the strongest between cell surface adhesion molecules, with a dissociation constant (*K*_*D*_) of 0.4–1.0 μM measured by surface plasmon resonance (59), and contributing to binding between T cells and APC with forces equivalent to those of integrins, as quantified by atomic force microscopy (60). Integrating this information with the inhibitory contribution toward signaling, it emerges that CD6 can possibly have a dual role, the first of which is to promote cellular adhesion, facilitating the TCR scanning of specific peptides. Upon antigen recognition and the formation of immunological synapses, CD6 can then adjust the strength of T cell activation through the attenuation of the signaling cascades. These features are also generic considering T cell activation as a whole, given that most APCs do express the ligand CD166. But the fact is that in different contexts with distinct APCs, possibly expressing varied levels of CD166 but also armed with different sets of costimulatory or co-inhibitory ligands for the many cell surface receptors of T cells, CD6 may impact differently on the signaling pathways and can eventually influence on the polarization of T cell subsets and responses, namely in the development of Th1 and Th17 sub-populations (61).

As for CD5, no APC-expressed ligand has been demonstrated so far; however, it was recently shown that it can serve as an alternative receptor for IL-6, leading to the activation of the transcription factor STAT3 (62). Although this observation was made in B1a cells, it nevertheless opens the perspective of IL-6 being able to promote Th17 responses when binding to T cell-expressed CD5.

## The Modulatory Role of CD6 During Thymocyte Development

Two recent reports on independent *Cd6* knockout models have finally confirmed that at the cellular level, the net contribution of CD6 to signaling is generally inhibitory (24, 25). Isolated CD4^+^ and CD8^+^ T cells from the mutant mice displayed significantly augmented activation upon anti-CD3 triggering than cells isolated from wild-type mice (24, 25), highlighting the inhibitory role of CD6 in T cell activation. It appears, however, that the strength of inhibition is milder than that of CD5.

**Figure 2:**
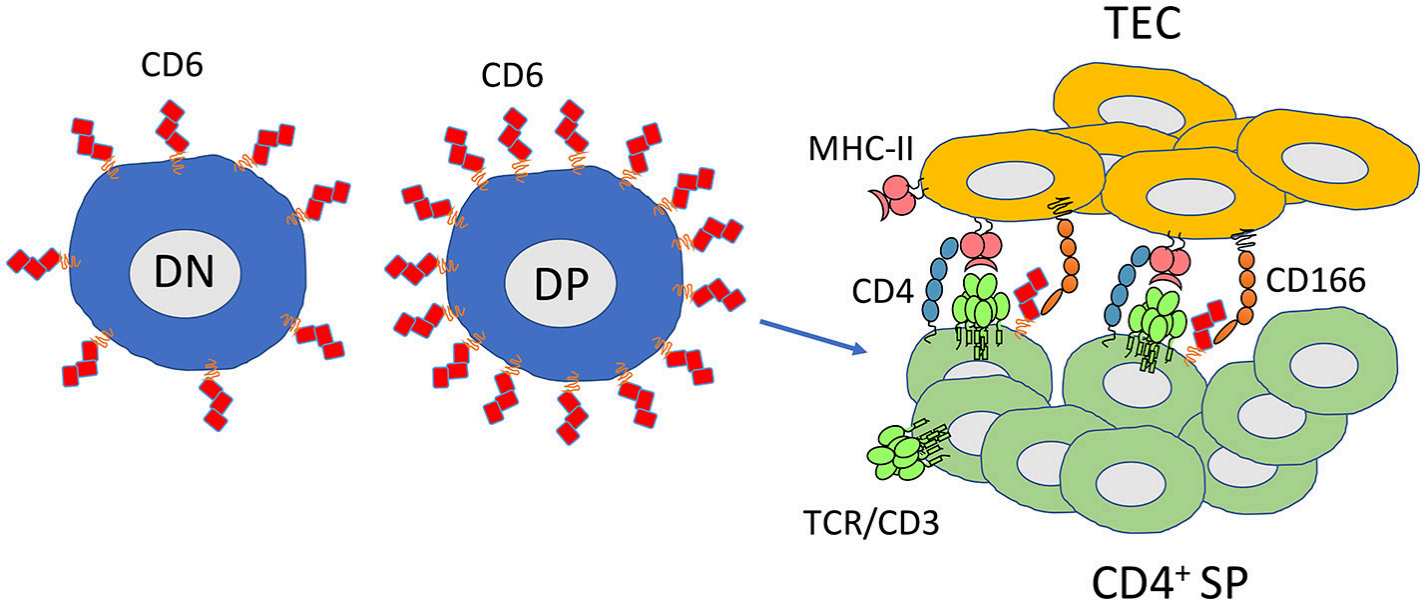
CD6 favors thymocyte differentiation and maturation and is required for selection of thymocytes with high-avidity TCRs. CD6 is expressed at all stages of thymocyte development. Its expression is increased at the double positive stage in cells that are assigned to maturation (CD69^high^). CD6 has an important role limiting the threshold for negative selection. The CD6-CD166 interaction promotes higher affinity TCR-MHC-II/peptide interactions contributing to CD4^+^ selection. CD6^−/−^ mice have an increased frequency of DP cells undergoing selection. Conversely, CD4^+^ SP and CD8^+^ SP have lower numbers or are less mature, respectively. DN, double negative thymocytes; DP, double positive thymocytes; CD4^+^ SP, single positive CD4 thymocytes; TEC, thymic epithelial cells.

The generation of *Cd6* knockout mouse models has been helpful in understanding the role of the protein during thymocyte selection. Although the frequency and total numbers of most cell populations in the periphery are identical comparing wild-type and CD6^−/−^ mice, in the thymus there seems to be a partial impairment in the transition from double-positive to single positive thymocytes (63). Namely, there is an increase in the percentage of DP thymocytes undergoing selection, which is accompanied by a decrease of CD4^+^ or CD8^+^ SP thymocytes that complete selection. Besides, the percentage of CD4^+^ SP thymocytes is reduced in these animals. The percentage of CD8^+^ SP cells is unaltered but a deeper look into that subpopulation revealed that, in *Cd6* knockout mice, the percentage of CD8^+^ immature cells was increased while that of CD8^+^ mature cells was decreased (63). In summary, CD6 is able to modulate signaling during T cell development as the lack of the molecule changes the threshold for negative selection in the thymus resulting in a reduced number of T cells that fully mature (Figure 2).

Similar studies had been conducted to assess the role of CD5 expression in development and how it affects the generation of either CD8^+^ or CD4^+^ T cell populations (18, 64). Despite the fact that the overall effect of CD5 expression in development was initially disguised by the large repertoire of expressed TCRs (65), the use of TCR-transgenic mice allowed to understand that CD5 acts as a negative regulator during T cell maturation (18, 64). In fact, a subsequent study encompassing different TCR-transgenic animal models clarified that the effect of CD5 is related to its levels of expression (22). In T cells with high-affinity TCRs (and consequent high expression of CD5), lack of CD5 markedly decreases positive selection, while increasing negative selection. In low-affinity TCR-expressing T cells, however, the loss of CD5 expression did not result in such significant changes (22).

CD6 is also able to impact on the efficiency of Tregs, a particular subset of T cells responsible for suppressing immune responses by inducing antigen tolerance. The ability of Tregs to downregulate both effector and helper T cells in response to self-antigens renders them important players in preventing autoimmune diseases. Tregs isolated from the spleen of *Cd6* knockout mice were shown to be less efficient in suppressing the proliferation of conventional T cells than those extracted from wild-type mice (63). Taking into consideration the many features common to both CD6 and CD5, this observation was somewhat surprising, as the opposite phenotype had been seen for *Cd5* knockout mice (66). Specifically, it was observed that Tregs from *Cd5* knockout mice were more efficient in their suppressive activity than cells from wild-type mice, consistent with CD5 being a negative regulator (66). The explanation for this discrepancy may be found in the global view of the system: CD4^+^ T cells from the spleen of *Cd5* knockout mice were described to express slightly more CD6; on the other hand, splenic CD4^+^ T cells from *Cd6* knockout mice tend to express less CD5 (63). Therefore, *Cd5* knockout mice ultimately display Tregs that are more able to repress immune responses whereas *Cd6* knockout mice, expressing less CD5 but also less CD6, are characterized by less efficient Tregs. This translates into less suppression of the immune system. In summary, when CD6 expression is increased directly or indirectly, the overall result seems to be a system more equipped to tone down immune responses.

## Ligand Binding in the Promotion of T Cell Migration vs. Subset Polarization

Mice with disrupted *Cd6* genes display differing responses in the two main inflammatory disease settings investigated so far. In the collagen-induced arthritis (CIA) model, *Cd6* knockout mice were characterized by earlier disease onset and increased clinical score as well as worsened hallmarks for the disease, namely IL-6 and TNF expression in the joints (63), whereas in a model of multiple sclerosis, experimental autoimmune encephalomyelitis (EAE), the absence of CD6 confers resistance to the demyelinating disorder (61). Although the mice strains were different and not the conventional usually used in the two different disease models, the disparate responses could again point to dual alternative roles of CD6 in T cell activation leading to divergent immune responses. However, when isolated and responding *in vitro* to antigenic or antibody-induced activation, CD6-negative cells were more responsive in both cases (61, 63). This observation again suggests that CD6 may have other roles than just its participation in signal transduction mechanisms.

Whereas, in the CIA model the augmented reactivity of CD6^−/−^ T cells is consistent with a cellular signaling inhibitory function for CD6, or an inhibitory CD6-mediated T cell response, in EAE the absence of CD6 was suggested, rather, to impair T cell migration through brain microvascular endothelial cells resulting in a lower infiltrate in the spinal cord of, among others, the autoreactive T cells. Although the experiments performed did not address leukocyte transmigration *in vivo* but actually used brain microvascular endothelial cells isolated from wild-type and knockout mice (61), these observations are concordant with a recent report where the re-engineered additional expression in CD6 molecules of the domain (d3) that interacts with CD166 significantly promotes T cell migration into the brain in a brain cancer model, where cancer endothelium upregulates CD166 expression (67). On the whole, a plausible explanation for the divergent impact of the lack of CD6 in the two models is that in CIA, CD6^neg^ cells are more autoreactive and inflict tissue damage whereas in EAE the lower T cell infiltration into the brain is the dominant effect.

The hypothesis that the lower EAE scores in the absence of CD6 are due to less transmigration is, however, in apparent contradiction with the interpretation that is given for the CD6-dependent corresponding human pathology: *CD6* is a susceptibility gene for multiple sclerosis (68), and individuals carrying a disease-related polymorphism at the locus rs17828933 within the first intron have increased levels of CD6Δd3 (69), a naturally occurring CD6 isoform that lacks the CD166-binding domain (70). Thus, in both mouse and human studies, the defective T cells are in disadvantage to cross the blood-brain barrier; but whereas in the mouse model the lack of cell infiltration is protective, in the human setting, paradoxically, the conditions are set to induce or aggravate the illness.

One main difference is that in the mouse the whole molecule is missing whereas in humans the full signaling potential is present. Furthermore, cells carrying the CD6Δd3 isoform are more reactive upon mAb challenge than cells expressing wild-type CD6. How can all these features be reconciled to explain the progression and severity of the disease? Unless we consider that the mouse and human studies are too different to be comparable, there is at present no simple answer to that question if only the signaling aspects of CD6 are taken into account. Therefore, a possible function of CD6 that should be considered is in fact its impact on the polarization of different T cell subsets in different disease conditions.

## Therapeutic CD6 mAbs

Bughani et al. have developed an anti-mouse CD6 mAb, mCD6D1, that recognizes the membrane distal domain of CD6 (d1), to ameliorate the incidence of EAE in C57BL/6 mice (26), while Li et al. have used a mouse anti-human CD6-d1 mAb, UMCD6, to reverse EAE progression in DBA/1 humanized mice (61). Itolizumab is a mouse mAb that also binds to CD6-d1 (71) and that has been found to be very efficient in treating autoimmune pathologies (72). Clinical trials for Itolizumab have been conducted to treat rheumatoid arthritis and psoriasis, yielding very encouraging results (73, 74). This antibody has already received approval from the Drugs Controller General of India as treatment for chronic plaque psoriasis in 2013, with treated patients presenting less proliferative T cells and decreased levels of pro-inflammatory cytokines in the serum (75). Despite the positive outcomes that are emerging from the use of this antibody, very little is known about its mechanism of action and how exactly it impacts on T cell signaling (72).

UMCD6 and Itolizumab recognize overlapping epitopes and can partly inhibit the binding of soluble CD166 to T cell-expressed CD6, although substantially less than anti-CD6-d3 (76). The structure of the CD6 extracellular region has only recently been solved (77), and it was speculated that antibody binding to CD6-d1 could perhaps hinder the ability of CD6 to interact with CD166 (26). However, in different experimental settings the effect of blocking of the direct interaction between T cell expressed-CD6 and APC-expressed CD166 by anti-CD6d1 mAbs, or even the reduction of T cell-APC conjugates, has produced contradictory results (70, 71, 78). Although it is possible that the presence of massive amounts of antibodies decorating the surface of T cells may actually reduce the number of T cell-APC conjugates, another plausible explanation is that the action of the reagents may have a direct effect on CD6-mediated signaling, or alternatively on CD6-mediated T cell polarization. The first case has been extensively documented *in vitro* and delivers very disparate results depending on the mAb clone used and conditions of cell culture and activation, ranging from significant activation to marked inhibition (79). As for the second, there is still insufficient documentation of *in vivo* studies to allow for any conclusions to be withdrawn for the time being; nevertheless, Itolizumab has proven efficacy to treat human diseases that are characterized by having Th17 polarization and concordantly, in human PBMC cultured in Th17 polarizing conditions, addition of Itolizumab seems to decrease the differentiation of CD4^+^ T cells into the Th17 sub-set and decrease the production of IL-17 (26).

## Concluding Remarks

Although perhaps counterintuitively at a first glance, signal inhibition relies heavily on intracellular tyrosine phosphorylation. Moreover, the phosphorylation of activation and inhibitory motifs can be catalyzed by exactly the same kinases and also be coincidental in time. Therefore, it cannot be unexpected that T cell-expressed inhibitory receptors functionally and physically associate with kinases that are known to be crucial to T cell activation. CD6 associates with LCK, FYN, ZAP70, and ITK, protein tyrosine kinases that are regarded as components of signaling progression, and yet, this complex molecule has been shown to bestow T cells with a strong inhibitory potential both *in vitro* as well as in *ex vivo* systems (34, 61, 63).

However, a straightforward categorization of CD6 as an inhibitor, or alternatively, as a co-stimulatory receptor, does not suffice to explain the apparent diversity of functions that the molecule displays in many different situations. CD6 seems to exhibit many features analogous to membrane-bound intracellular adaptors, such as LAT and PAG, as it has multiple tyrosine residues on its cytoplasmic domain that once phosphorylated can couple to a range of different enzymes and adaptors, possibly feeding into multiple parallel signaling pathways. Perhaps this can explain the antagonistic roles attributed to the molecule in different experimental setups. On the other hand, the fact that CD6 establishes robust extracellular interactions with APC-expressed ligands strongly favors the adhesion between T cells and APCs and unequivocally promotes T cell activation. Whether binding to CD166 can, alternatively in different situations, promote T cell activation or repression, or impact on T cell subset polarization requires further investigation. CD6 is being recognized as an important target for therapy against several autoimmune diseases and the use of therapeutic CD6 mAbs is steadily increasing. A major challenge facing ahead is to understand how these reagents can regulate CD6 function to be able to devise the most appropriate treatment for human disease.

## Author Contributions

CMG, SNH, RFS, and AMC wrote the manuscript. CMG and AMC designed the figures.

### Conflict of Interest Statement

The authors declare that the research was conducted in the absence of any commercial or financial relationships that could be construed as a potential conflict of interest.
